# Supplementary material for: Highly efficient current-induced domain wall motion in a room temperature van der Waals magnet
Source: Nat Commun. 2025 Nov 27;16:10790. doi: 10.1038/s41467-025-66637-4 (PMC12663122; doi:10.1038/s41467-025-66637-4)
Supplement: Supplementary file 1 — Supplementary Information [file 41467_2025_66637_MOESM1_ESM.pdf]

Supplementary Information

Highly efficient current-induced domain wall motion in a room temperature van der Waals magnet

Yicheng Guan[1, †], Yufeng Wu[1, †], Yan Zhang[1], Jae-Chun Jeon[1], Wenjie Zhang[1], Ke Xiao[1] and Stuart S. P. Parkin[1, *]

1. Max-Planck Institute for Microstructure Physics, 06120 Halle (Saale), Germany

† These authors contribute equally: Yicheng Guan, Yufeng Wu

* E-mail: stuart.parkin@mpi-halle.mpg.de

This file includes:

- Supplementary Note 1-4
- Fig. S1-S10
- Table S1-S3
- References for SI

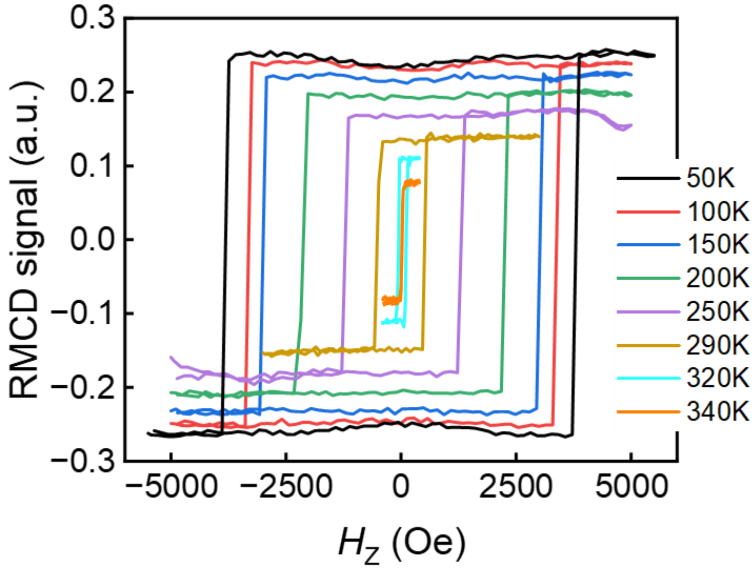

Figure S1. Magnetization versus out of plane magnetic field hysteresis loops for a Fe_3GaTe_2 flake at various temperatures. (a) Reflective magnetic circular dichroism (RMCD) measurements of hysteresis loops for a Fe_3GaTe_2 (FGaT) flake with a thickness of ~25 nm at various temperatures.

Supplementary Note 1. In-plane field dependence of the CIDWM in FGaT.

In FGaT, the presence of a bulk Dzyaloshinskii-Moriya interaction (DMI) has been established by earlier studies that show the existence of Néel type skyrmions[1-3]. Similar to its sister material Fe_3GeTe_2, the DMI in FGaT originates from a broken inversion symmetry due to Fe vacancies in the van der Waals layers and additional Fe atoms in the van der Waals gaps[2]. Such a DMI leads to Néel type domain walls in FGaT, which is further confirmed from the in-plane longitudinal field H_X dependence of the current-induced domain wall velocity v. Fig. S2 shows dome-like v- H_X curves that are offset to positive and negative fields for up/down and down/up domain walls. This behavior is a typical feature of Néel type domain walls and is well described using the well-known one-dimensional (1D) model[4,5] with parameters obtained from our measurements (see Table S1).

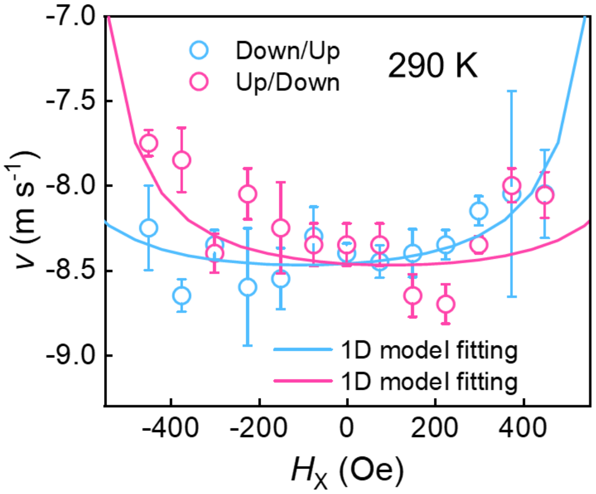

Figure S2. In-plane longitudinal field, H_X, dependence of the domain wall velocity, v, in a FGaT racetrack device at 290 K. Pink and light blue open circles correspond to up/down and down/up domain wall configurations, respectively. Solid lines are fits to the 1-D domain wall model. Current pulses with a current density of 3.2 MA cm^{-2} and a pulse length of 5 ns are used. All error bars correspond to 1SD.

Domain wall width (nm)	Damping parameter	Non-adiabatic term	Shape anisotropy field (Oe)	Effective DMI field (Oe)	Saturation magnetization (emu cm^{-3})
1.7	0.13	0.5	1200	180	240

Table S1. Fitting parameters used in the 1D domain wall model fit in Fig. S2.

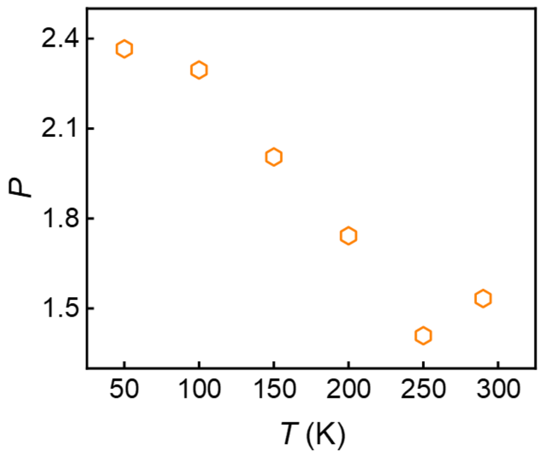

Figure S3. Temperature dependence of the calculated spin polarization, *P*, in the absence of the non-adiabatic term. The polarization rate *P* is obtained using the 1D model of STT-driven domain wall motion with a zero non-adiabatic term.

Supplementary Note 2. Numerical fitting based on the modified Blonder–Tinkham–Klapwijk (BTK) model for normalized conductance to bias voltage curve.

We have carried out the numerical fitting based on the modified BTK model to our normalized conductance to bias voltage curve data in Fig. S4. The fitting parameters used are shown in Table S2. The two dips at bias voltage of ~ ± 2 meV are not considered for the fitting since there exists no superconducting gap that can account for the position of these dips. *P* value obtained from the fitting (~ 0.38) shows a similar value to that obtained in our main text using the approximation (~ 0.415). We have also conducted the BTK fitting using a modified two-gap model for the 2H-$NbSe_2$ as reported by former literatures[6,7], which also gives a similar *P* value of ~0.42 (see Fig. S4b and Table S3).

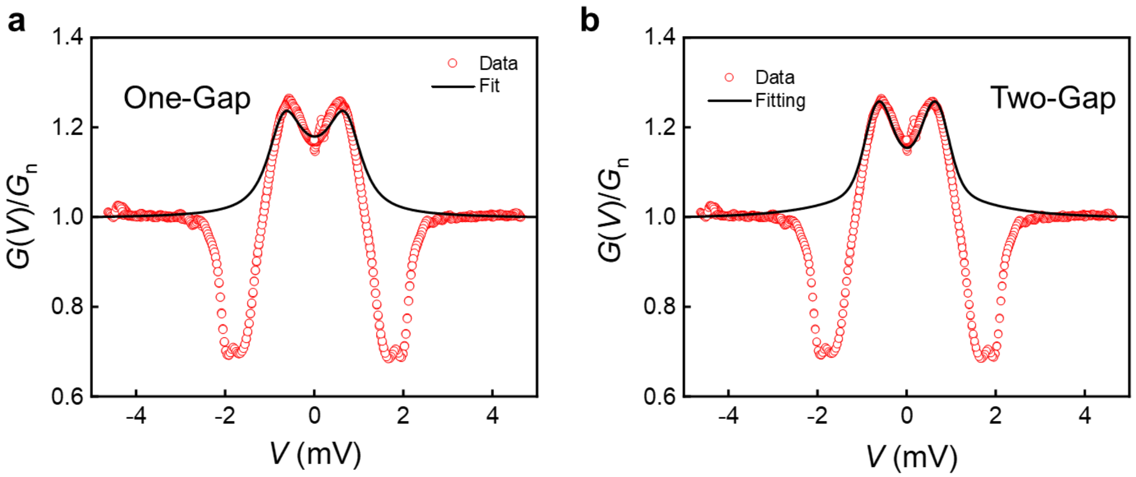

Figure S4. Fitting of the normalized conductance to bias voltage curve from the superconducting point contact measurement using a one-gap BTK model (a) and a two-gap BTK model (b).

Δ (meV)	*Γ* (meV)	*Z*	*P*	*T*
0.7	0.01	0.15	0.38	2

Table S2. Fitting parameters used for the modified one-gap BTK model.

Δ_1 (meV)	Γ_1 (meV)	Z_1	Δ_2 (meV)	Γ_2 (meV)	Z_2	*P*	*T* (K)
1.1	0.65	0	0.6	0.4	0.9	0.42	2

Table S3. Fitting parameters used for the modified two-gap BTK model.

Supplementary Note 3. Transport measurement for determining the effective uniaxial magnetic anisotropy energy in 25 nm Fe_3GaTe_2 flakes.

In order to determine the effective uniaxial magnetic anisotropy energy, we fabricated the six-terminal Hall bar device to measure the anomalous Hall signal with exterior magnetic field at various azimuthal angles. Fig. S5 shows the temperature-dependent anomalous Hall measurement with an out-of-plane (OOP) field. The device has a dimension of 10 μm long and 5 μm wide. By fitting the saturated anomalous resistance with the same equation used in main text as $R_{\mathrm{XY}}(T) = R_{\mathrm{XY}}^{0} \times (1 - T/T_{\mathrm{c}})^{\tau}$, a T_{c} of 345 K can be achieved, same with those obtained from RMCD measurement. It is worth noticing, in the longitudinal resistance, similar to previous research, a kondo-like behavior is observed[8]. A fitting equation of $R(T) = R_0 - R_{\mathrm{s}} \ln(T) + R_e T^{\frac{1}{2}} + R_p T$ is used, where R_0 is the residual resistance, R_{s} is from the electron-spin scattering, R_e from the electron-electron scattering and R_p from the electron-phonon scattering[9]. The fitting gives comparable values of R_{s} (– 6.7 ± 0.12) and R_e (– 8.1 ± 0.07), showing the important role of spin disorder scattering in FGaT. The upper rising temperature at 50 K is in good correspondence to the increase of both Gibert damping and non-adiabatic terms at the same temperature. In the 25 nm FGaT flake, an anomalous Hall angle as large as 2 % can be observed at low temperature.

The exterior magnetic field is rotated from OOP direction to in-plane direction to determine the uniaxial magnetic anisotropy energy K_u^{eff}. Typical set-up and R_{XY} hysteresis loop are shown in Fig. S6a. In order to prevent the influence of anisotropic magnetoresistance, the field is applied in transverse direction (*y-z* plane). By varying the azimuthal angle of exterior field θ_{B}, the azimuthal angle of magnetization in FGaT can be expressed as $\cos(\theta_M) = R_{\mathrm{XY}}(B)/R_{\mathrm{XY}}(0)$. By following the method described in Ref. 10 through calculating the energy minimum, the effective uniaxial anisotropy field H_K^{eff} can be obtained from linear fitting of $\sin(\theta_B - \theta_M)$ - $\sin(2\theta_M)$ curve[10]. By taking a literature value of M_{S} = 417 emu cm^{-3} at 3 K and the equation of $M(T) = M_0 \times (1 - T/345)^{0.3}$, M_{S} at each temperature is calculated and thus the K_u^{eff} is calculated using the equation of $K_u^{\mathrm{eff}} = H_K^{\mathrm{eff}} \times M_{\mathrm{S}}/2$.

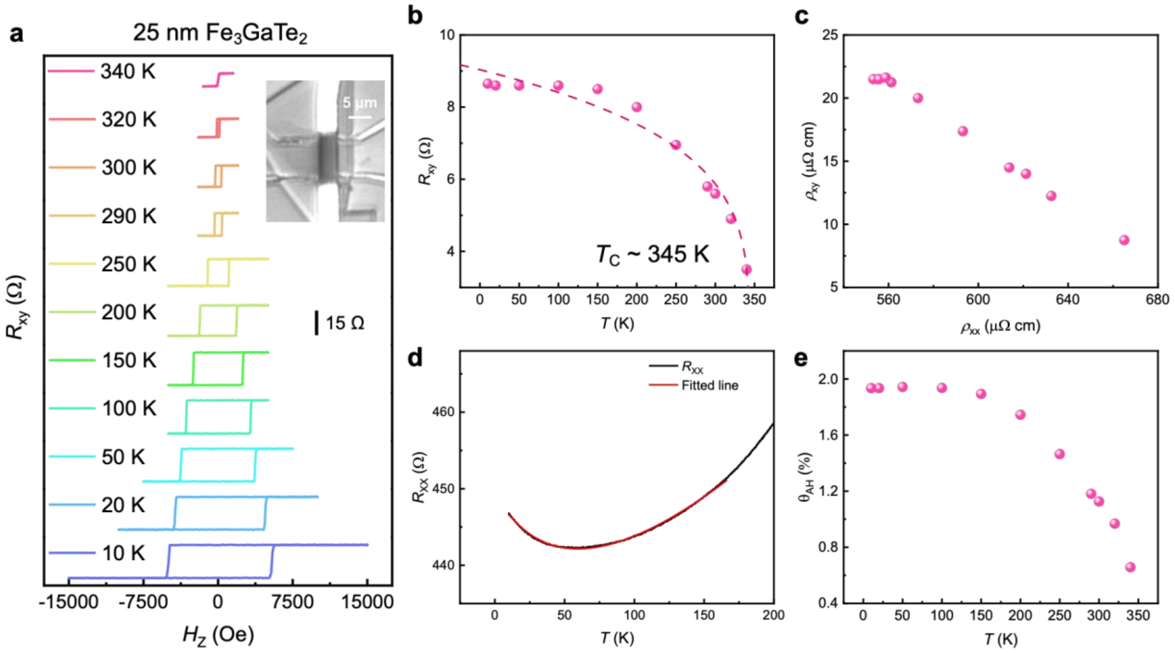

Figure S5. Temperature-dependent Hall measurement of Fe_3GaTe_2. (a) The hysteresis loop of anomalous Hall resistance R_{XY} by exterior magnetic field H_Z at various temperatures. The summarized anomalous Hall resistance R_{XY} (b), longitudinal resistance R_{XX} (d) and anomalous Hall angle θ_{AH} (e) plotted as a function of temperature. (c) The calculated anomalous Hall resistivity ρ_{XY} plotted as a function of sheet resistivity ρ_{XX}. The fitted line in (d) follows the equation described in Supplementary Note 1.

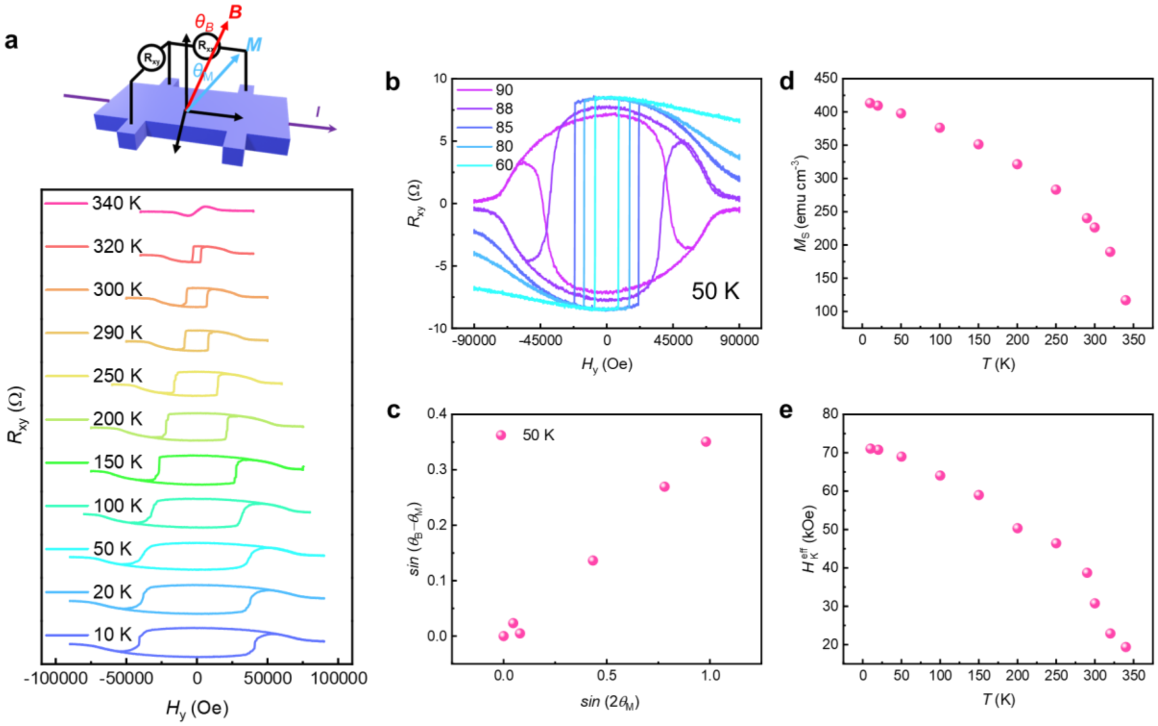

Figure S6. Transport measurement to determine the effective uniaxial anisotropy energy of Fe_3GaTe_2. (a) The experimental set-up and the hysteresis loop of anomalous Hall resistance R_{XY} by the exterior in-plane transverse field H_Y at various temperatures. (b) The hysteresis loop of R_{XY} by exterior field applied at different azimuthal angles (θ_B) at 50 K. (c) The calculated sine value of azimuthal angle difference between exterior field and magnetization ($\theta_B - \theta_M$) plotted as a function of the sine value of $2\theta_M$ to determine the effective uniaxial anisotropy field. The temperature dependence of the calculated saturation magnetization M_s (d) and effective uniaxial anisotropy field H_K^{eff} (e).

Supplementary Note 4. Spin-transfer torque efficiency calculated from DW mobility

In the main text, we have measured the domain wall mobility induced by current (Fig. 1e), as well as the domain wall mobility by field (Fig. 3b), namely, mobility-J and mobility-H, respectively. A direct comparison between these two values are carried out to calculate the spin-transfer torque efficiency (mobility-J/mobility-H)[11]. The results are shown in Fig. S7. With decreasing the temperature, the STT efficiency increases. All error bars in Fig. S7 correspond to 1SD.

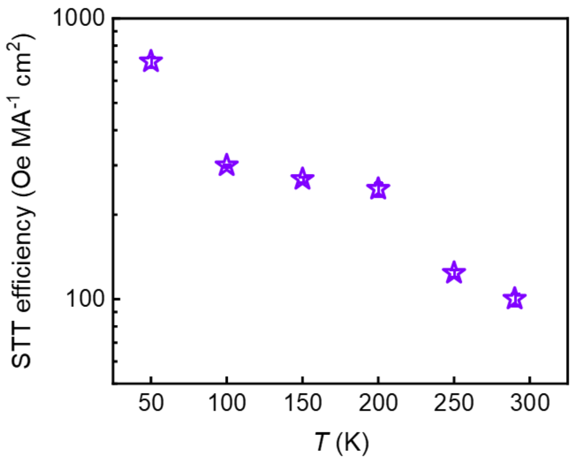

Figure S7. The temperature dependence of spin-transfer torque efficiency in FGaT.

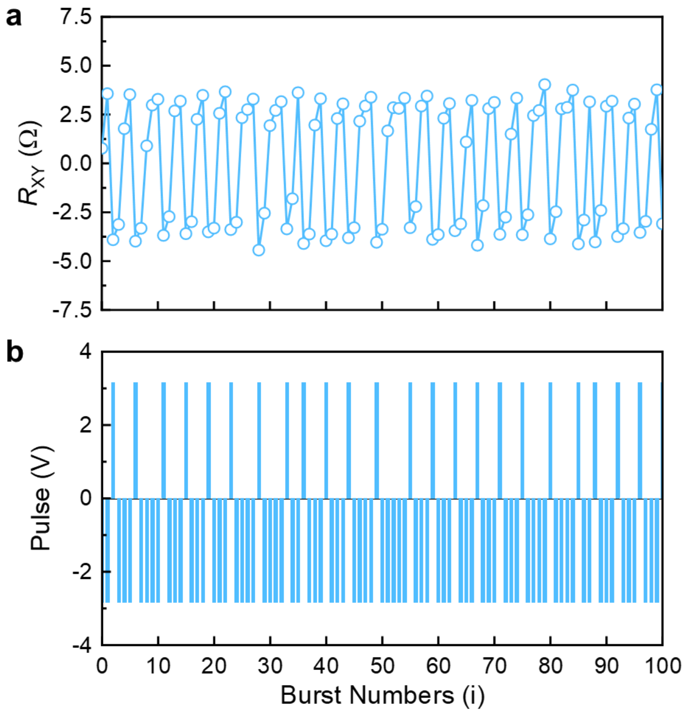

Figure S8. Electrical manipulation of domain wall in a Racetrack memristor device S2. (a) The evolution of anomalous Hall resistance (R_{XY}) via injecting a burst of 20 voltage pulses with amplitude of ± 3.6 V and pulse length of 5 ns. (b). The corresponding pulse trains.

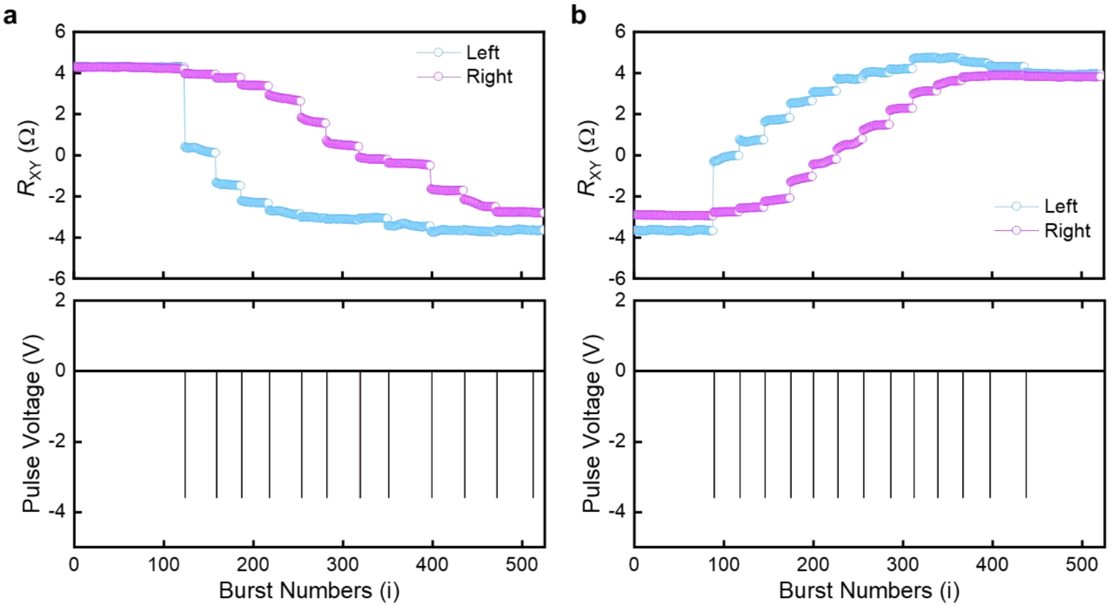

Figure S9. Electrical detection of single domain wall across multiple Hall bar detectors S3. The electrical signals to detect one single DW moving across two hall bars which are placed 10 μm away from each other. Trains of 20 voltage pules with amplitude of - 3.6 V and length of 5 ns are used to drive the DW. A ↑↓ DW (a) and ↓↑ DW (b) is created and then moves from left to right. The corresponding trains of injected voltage pulses are shown below.

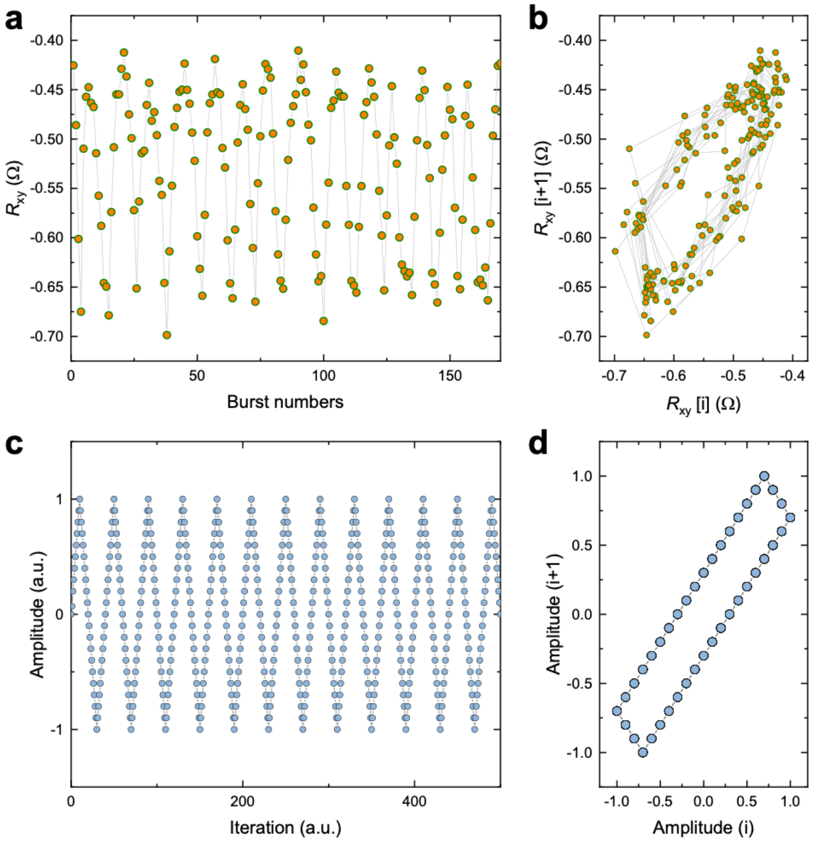

Figure S10. (a) The evolution of anomalous Hall resistance in racetrack memristors with burst numbers over 150, with 5 pulses in one burst. (b) The corresponding static phase space plot of the R_{XY}. (c) Simulated time (iteration)-series signal from an ideal racetrack without pinning. (d) The static phase plot of the perfect linear current induced domain wall motion.

References

1 Li, Z. *et al.* Room-temperature sub-100 nm Néel-type skyrmions in non-stoichiometric van der Waals ferromagnet $Fe_{3-x}GaTe_2$ with ultrafast laser writability. *Nat. Commun.* **15**, 1017 (2024).

2 Saha, R., Meyerheim, H. L., Göbel, B., Mertig, I. & Parkin, S. S. P. High-temperature Néel skyrmions in Fe_3GaTe_2 stabilized by Fe intercalation into the van der Waals gap. *npj Spintr.* **2**, 21 (2024).

3 Zhang, C. *et al.* Above-room-temperature chiral skyrmion lattice and Dzyaloshinskii–Moriya interaction in a van der Waals ferromagnet $Fe_{3-x}GaTe_2$. *Nat. Commun.* **15**, 4472 (2024).

4 Zhang, W. *et al.* Current-induced domain wall motion in a van der Waals ferromagnet Fe_3GeTe_2. *Nat. Commun.* **15**, 4851 (2024).

5 Filippou, P. C. *et al.* Chiral domain wall motion in unit-cell thick perpendicularly magnetized Heusler films prepared by chemical templating. *Nat. Commun.* **9**, 4653 (2018).

6 Guillamón, I. *et al.* Superconducting density of states and vortex cores of 2H-NbS_2. *Phys. Rev. Lett.* **101**, 166407 (2008).

7 Noat, Y. *et al.* Quasiparticle spectra of 2 H-$NbSe_2$: Two-band superconductivity and the role of tunneling selectivity. *Phys. Rev. B* **92**, 134510 (2015).

8 Wang, M. *et al.* Hard ferromagnetism in van der Waals Fe_3GaTe_2 nanoflake down to monolayer. *npj 2D Materials and Applications* **8**, 22 (2024).

9 Zhang, J. *et al.* Kondo-like transport and its correlation with the spin-glass phase in perovskite manganites. *Physical Review B—Condensed Matter and Materials Physics* **72**, 054410 (2005).

10 Deng, Y. *et al.* Room-Temperature Highly Efficient Nonvolatile Magnetization Switching by Current in van der Waals Fe_3GaTe_2 Devices. *Nano Letters* **24**, 9302-9310 (2024).

11 Wang, Q. *et al.* Magnetism modulation in $Co_3Sn_2S_2$ by current-assisted domain wall motion. *Nat. Electro.* **6**, 119-125 (2023).
